# Potential therapeutic option for EGFR-mutant small cell lung cancer transformation: a case report and literature review

**DOI:** 10.3389/fimmu.2024.1439033

**Published:** 2024-08-21

**Authors:** Xiaoxuan Li, Xinchi Luan, Mengqi Zhang, Rui Wang, Jing Guo, Jing Lv, Wensheng Qiu, Shufen Zhao

**Affiliations:** Department of Oncology, The Affiliated Hospital of Qingdao University, Qingdao, Shandong, China

**Keywords:** non-small cell lung cancer (NSCLC), small cell lung cancer (SCLC), pathological transformation, EGFR exon 19 deletion (19 del), combination therapy, case report

## Abstract

Transformation from non-small cell lung cancer (NSCLC) to small cell lung cancer (SCLC) is rare and is associated with poor prognosis. However, the standard treatment protocols for patients with SCLC transformation remain unknown. Here, we report the case of a patient with advanced EGFR exon 19 deletion (19del) NSCLC who underwent SCLC transformation during targeted therapy. Biopsies and genetic testing were performed to adjust treatment regimens accordingly. The patient responded favorably to a combined treatment regimen comprising etoposide plus cisplatin chemotherapy and adebrelimab plus osimertinib. This case highlights the critical importance of acknowledging tumor heterogeneity in clinical decision-making and identifying potentially effective treatment options for patients with SCLC transformation. Additionally, we reviewed cases of the transformation of NSCLC to SCLC from 2017 to 2023.

## Introduction

The management of non-small cell lung cancer (NSCLC) and small cell lung cancer (SCLC) is a critical area of investigation in the field of oncology. NSCLC, which accounts for 80-85% of all lung cancers, plays a significant role in targeted therapy (1, 2). EGFR exon 19 deletion (19del) is a common genetic alteration observed in patients with advanced NSCLC (3). When treated with EGFR-tyrosine kinase inhibitor (TKI), some EGFR-mutated NSCLC patients may undergo rare pathological transformations to SCLC (4), which is an important mechanism for resistance to EGFR-TKI treatment. Several studies have reported that NSCLC-derived SCLCs exhibit clinical features similar to primary SCLCs (5). However, for patients who undergo transformation from NSCLC to SCLC, chemotherapy provides only short-term effectiveness and leads to poor prognosis, with a median overall survival (OS) of less than 1 year (6). Therefore, the timely identification and development of effective treatment strategies are crucial. Although SCLC transformation in NSCLC patients has been documented in the literature (**Table 1**), there is no clear consensus on the optimal treatment regimen for these patients.

**Table 1:** Summary of cases of small cell lung cancer transformed from non-small cell lung cancer (2017 to 2023).

Case Number	Report Year	Age (years)	Sex	Country	Smoking status	Mutational status of tumor sample	Medication taken before the transition	Medication taken after the transition	CNS metastasis	OS after transformation	OS	Reference
1	2017	75	Male	Japan	Smoker	Negative	Docetaxel and bevacizumab followed by nivolumab	Amurubicin	NM	About 2 months	About 8 months	(7)
2	2018	62	Male	Japan	Smoker	ALK rearrangement	PC, bevacizumab, followed by alectinib	Alectinib followed by EP and then AMR, nivolumab, and irinotecan	Yes	About 8 months	About 4 years	(8)
3	2018	65	Male	USA	Smoker	Negative	PC and then nivolumab	EC	NM	NA	NA	(9)
4	2018	68	Male	USA	NM	NM	TC and pembrolizumab	EC	NM	NA	NA	(9)
5	2018	38	Male	China	Never-smoker	EGFR exon 21 L858R	PP followed by erlotinib	EP	Yes	NA	NA	(10)
6	2018	69	Male	Japan	NM	EGFR 19del	Erlotinib and pemetrexed plus bevacizumab	IP followed by afatinib and then osimertinib	Yes	NA	NA	(11)
7	2019	67	Female	USA	Smoker	TP53, RB1	carboplatin and P TX and then nivolumab	EC and then paclitaxel	NM	About 11 months	About 4 years	(12)
8	2019	75	Female	USA	Smoker	KRAS G12C, TP53	Nivolumab	EC and then nivolumab and then ipilimumab and then irinotecan	NM	About 16 months	About 5.5 years	(12)
9	2019	66	Male	Japan	Smoker	EGFR	TC and bevacizumab and then pembrolizumab	EC and then amrubicin	NM	About 5 months	About 12 months	(13)
10	2019	70	Female	Israel	Smoker	TP53	Nivolumab	NM	NM	NA	NA	(14)
11	2019	75	Male	Israel	Smoker	TP53	Nivolumab	EC	NM	About 13 months	About 31 months	(14)
12	2020	65	Male	Japan	Smoker	Strongly positive for PD-L1	Pembrolizumab	IP and then Amrubicin	No	About 17 months	NM	(15)
13	2020	69	Male	China	Smoker	TP53 mutation; R342* nonsense mutation	Pembrolizumab	EC	NM	NA	NA	(16)
14	2020	60	Female	USA	Smoker	TP53, CDKN2A R58, PIK3CA E545K mutation; SOX2 PIK3CA, CCND2, CCND3, MYCL1, CSF3R, FGF23, FGF6, C17orf39, KDM5A, PRKCI, TERC, VEGF amp	carboplatin and gemcitabine and then nivolumab	EC	NM	About 14 months	About 39 months	(17)
15	2020	62	Male	Japan	NM	High PD-L1 (70%) expression, TP53 inactivation and RB1 loss	IP and then pembrolizumab	EP	NM	NA	NA	(18)
16	2020	56	Male	China	Smoker	EGFR 19del, EGFR amp, RB1, TP53, MSH6, PMS2 amp; PD-L1 (–); TMB of 15.32 Muts/Mb; MSS	Icotinib	EC followed by docetaxel, sequential icotinib, irinotecan, anlotinib, and pabolizumab	NM	About 9 months	About 15 months	(19)
17	2020	68	Male	Japan	Smoker	EGFR 19 del, T790M	Osimertinib followed by erlotinib and then osimertinib and then carboplatin, paclitaxel, docetaxel, and pemetrexed and then S-1 monotherapy	EC	NM	NA	NA	(20)
18	2021	63	Female	Italy	Never-smoker	EGFR 19del and T790M, TP53	Gefitinib followed by osimertinib	Platinum–etoposide doublet followed by paclitaxel and whole-brain radiotherapy	Yes	NA	NA	(21)
19	2021	64	Male	Japan	Smoker	NM	CBDCA and docetaxel and then nivolumab	IC, AMR, nab-paclitaxel	NM	NA	NA	(22)
20	2021	70	Male	Japan	Smoker	NM	TC and then nivolumab	Etoposide	NM	NA	NA	(22)
21	2021	74	Female	Japan	Never-smoker	NM	TC followed by vinorelbine and then nivolumab and then atezolizumab	AMR	NM	NA	NA	(22)
22	2021	43	Male	China	Never-smoker	EGFR 19del and high PD-L1 (80.9%) expression	Gefitinib followed by 8 cycles of pembrolizumab plus pemetrexed and then osimertinib	EP followed by anlotinib plus gefitinib and then EC plus durvalumab	NM	About 20 months	About 7 years	(23)
23	2021	57	Male	China	Smoker	EGFR 19 del, EGFR exon20p, MYC amp, RB1, TP53, T790M, EGFR amp	Gefitinib	EC followed by irinotecan and nedaplatin plus icotinib	Yes	NA	NA	(24)
24	2022	84	NM	China	Smoker	EGFR exon 21 L858R	Osimertinib	Durvalumab and EC	Yes	NA	NA	(25)
25	2022	63	Female	China	Never-smoker	EGFR	Gefitinib	Refuse treatment	NM	About 12 months	About 22 months	(26)
26	2022	50	Male	China	Smoker	EGFR 19del and T790M	Erlotinib followed by toripalimab plus PC	EC followed by osimertinib	Yes	NA	NA	(27)
27	2022	44	Male	China	NM	EGFR 19del, TP53 Y220H, RB1 F755V	Icotinib	Combined radioactive particle implantation and 6 cycles of IP chemotherapy followed by paclitaxel plus cisplatin and then apatinib followed by GP	Yes	NM	About 3 years	(28)
28	2023	56	Male	China	Smoker	Negative	Sugemalimab (neoadjuvant with chemotherapy); Sugemalimab (consolidation therapy)	EP	NM	About 6 months	About 14 months	(29)
29	2023	58	Female	China	NM	EGFR L858R, T790M, TP53, RB1	Osimertinib	EP followed by osimertinib in combination with EP, and then osimertinib and anlotinib	Yes	About 11 months	About 35 months	(30)
30	2023	43	Female	France	Smoker	TP53	Alectinib	Pralsetinib followed by EC and then TC	Yes	NA	NA	(31)
31	2023	30	Female	China	Never-smoker	KIF5B-RET fusion	No treatment during pregnancy and then selpercatinib	HS-10365 followed by EC	Yes	NA	NA	(32)
32	2023	52	Male	China	Smoker	EML4-ALK fusion, KRAS G12D, TP53, RB1, PIK3CA, ALK V1180L; TMB of 11.3 muts/Mb; MSS	Ensartinib followed by alectinib	EP followed by lorlatinib	Yes	NA	NA	(2)
33	2023	77	Male	China	NM	EML4-ALK fusion, CRKL amp, VEGFR1 amp, loss of RB1; TMB of 2.23 muts/Mb; MSS	Alectinib followed by radiotherapy	Atezolizumab plus EC, remaining alectinib	Yes	About 9 months	About 21 months	(33)
Our case	2024	71	Female	China	Never-smoker	EGFR 19 del	Osimertinib	Anlotinib and aumolertinib followed by EP plus adebrelimab and then osimertinib, EP, plus adebrelimab	No	NA	NA	

*The asterisk means termination codon.

amp, amplification; AMR, ceritinib, alectinib, and amrubicin; CNS, central nervous system; EC, etoposide plus carboplatin; EP, etoposide plus cisplatin; GP, gemcitabine plus cisplatin; IC, irinotecan plus carboplatin; IP, irinotecan plus cisplatin; MSS, microsatellite stability; NA, not applicable; NM, Not mentioned; OS, overall survival; PC, pemetrexed plus carboplatin; PP, pemetrexed plus cisplatin; TC, paclitaxel plus carboplatin; TMB, tumor mutational burden; 19del, exon19 deletion.

Here, we describe the case of a patient with advanced NSCLC with EGFR 19del who underwent pathological transformation from NSCLC to SCLC. Repeated biopsies and next-generation sequencing (NGS) tests, along with clinical disease evolution, have underscored tumor heterogeneity. These findings indicate that multimodal treatment, including chemotherapy, targeted therapy, and immunotherapy, may be a viable therapeutic strategy for this specific patient group.

## Case presentation

### Diagnosis and initial treatment response

**Figure 1:**
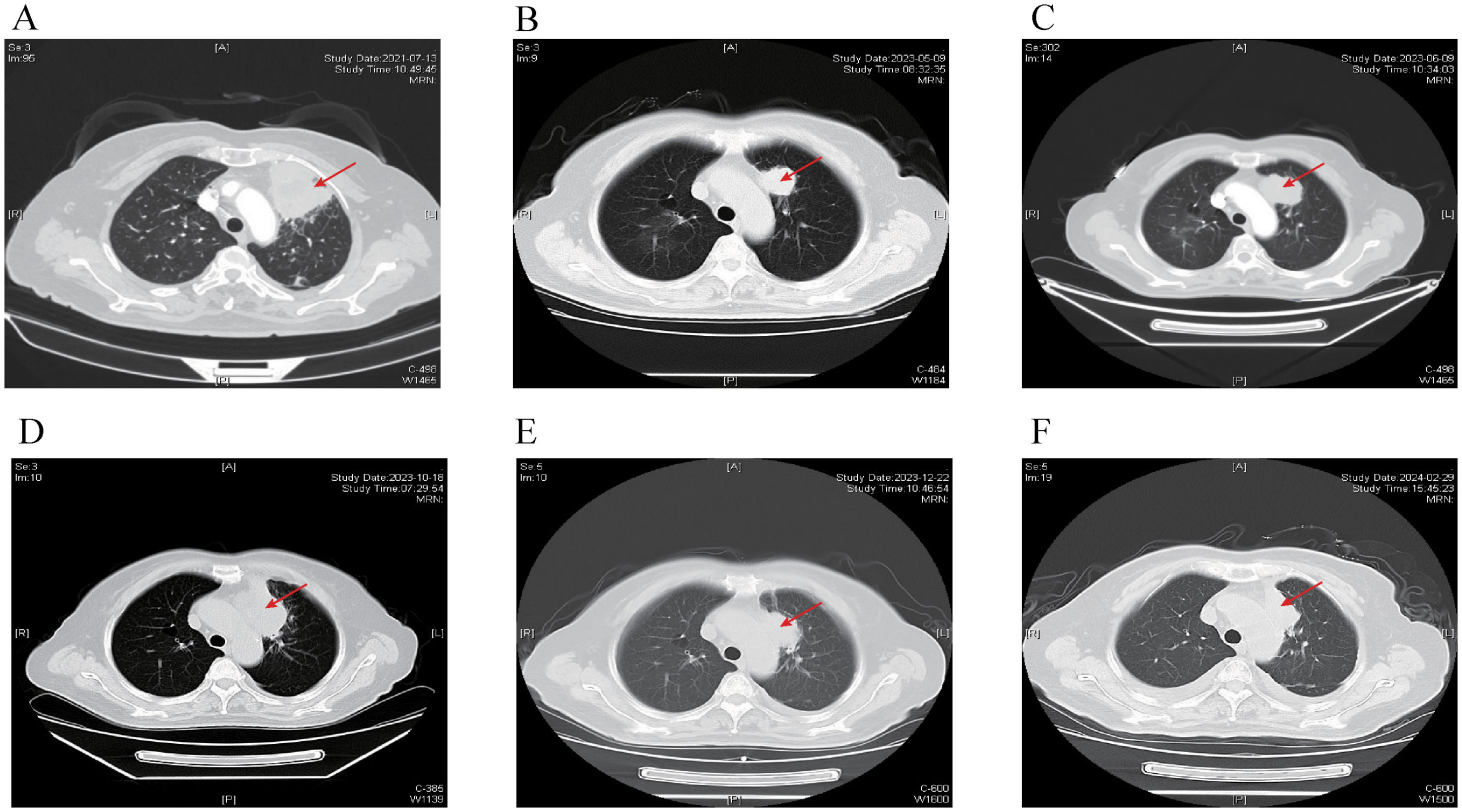
Chest CT scans at different time points. The red arrow indicates primary lesion in the left upper lobe of the lung. **(A)** Chest CT scan of baseline. **(B)** Chest CT scan of best response PR after first-line treatment with Osimertinib. **(C)** Chest CT scan showing progression after 24 months of Osimertinib. **(D)** Chest CT scan showing progression after 4 months of Anlotinib and Aumolertinib. **(E)** Chest CT scan showing reduction in the LUL lesion after 2 cycles of EP chemotherapy plus Adebrelimab. **(F)** Chest CT scan showing regression in the LUL lesion after fourth-line treatment with Osimertinib in addition to the existing chemotherapy and immunotherapy regimen. CT, computed tomography; PR, partial response; LUL, left upper lobe; EP, etoposide plus cisplatin.

**Figure 2:**
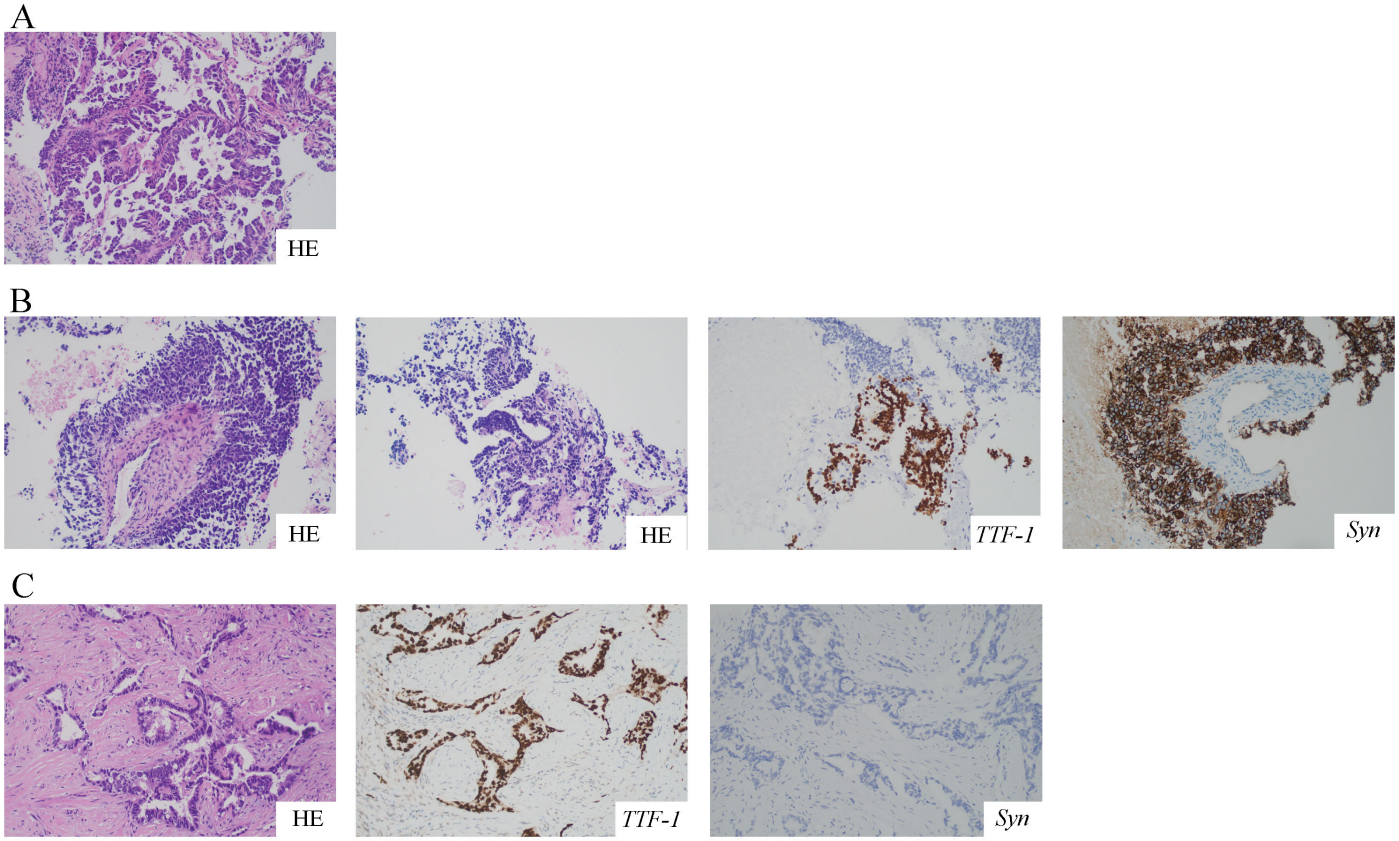
HE and IHC staining of the tumor at different time points. All pictures were taken at a 200-fold magnification using a light microscope. **(A)** Biopsy specimen of LUL revealed poorly differentiated lung adenocarcinoma with HE staining. **(B)** The second biopsy of LUL revealed mixed histology of adenocarcinoma and SCLC with HE and IHC staining for *TTF-1* and *Syn*. **(C)** The third biopsy of the right cervical lymph node revealed poorly differentiated adenocarcinoma with HE and IHC staining for *TTF-1* and *Syn*. HE, hematoxylin and eosin; IHC, immunohistochemistry; LUL, left upper lobe; SCLC, small cell lung cancer; *TTF-1*, thyroid transcription factor-1; *Syn*, synaptophysin.

A 68-year-old female was admitted to the hospital on July 12, 2021, because of cough and expectoration for 2 months. The patient had no history of smoking or cancer history. Contrast-enhanced chest computed tomography (CT) revealed a mass in the upper lobe of the left lung, along with multiple small nodules in both lower lobes and enlarged mediastinal and hilar lymph nodes. Moreover, pleural thickening and pleural effusion were observed (**Figure 1A**). Biopsy of the enlarged lesion in the left upper lobe (LUL) revealed poorly differentiated adenocarcinoma of the lung (**Figure 2A**). 14-gene panel testing identified an EGFR 19del mutation (**Table 2**). The patient was diagnosed with stage IV lung adenocarcinoma with EGFR 19del. The patient achieved partial response (PR) after first-line treatment with osimertinib (**Figure 1B**). Progression-free survival (PFS) after the first-line treatment was 24 months.

**Table 2:** Overview of patient’s multiple next-generation sequencing results.

Gene name	Mutations	Mutation frequency/copy number		
		LUL before treatment	LUL after treatment	Right cervical lymph node
EGFR	p.L747_A755delinsSKD 19del	26.10%	45.85%	8.07%
TP53	p.P278T exon8 missense mutation		83.52%	33.48%
RB1	p.E464* exon15 nonsense mutation		80.16%	38.84%
EGFR	gene amplification		6.6-fold	NA
KIT	gene amplification		4.1-fold	NA
MDM4	gene amplification		NA	6.0-fold

19del, exon19 deletion; LUL, left upper lobe; NA, not applicable.

### Disease progression and SCLC transformation

**Figure 3:**
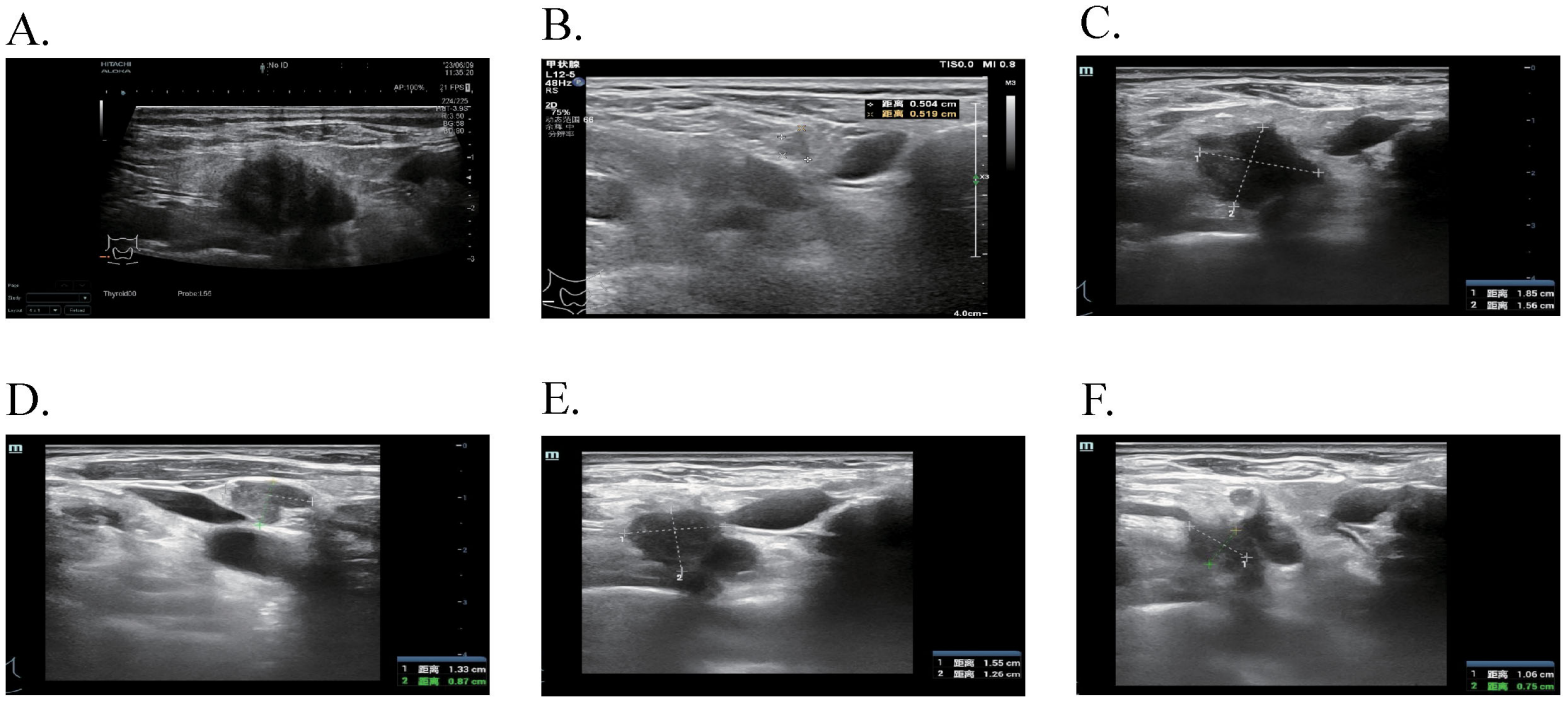
The ultrasound features of cervical lymph nodes at different time points. **(A)** After 24 months of Osimertinib, enlarged lymph nodes were observed in the IV region of the right neck, with a maximum size of 2.3 × 1.7 cm. **(B)** Following dual-targeted therapy, a previously enlarged lymph node in the IV region of the right neck reduced to 0.5 × 0.5 cm. **(C, D)** After 2 cycles of EP chemotherapy plus Adebrelimab, increased and enlarged lymph nodes were detected in the right neck IV area, with the largest measuring 1.9 × 1.6 cm and 1.3 × 0.9 cm. **(E, F)** With the addition of Osimertinib to the existing chemotherapy and immunotherapy regimen, the enlarged lymph nodes in the right neck IV area measured 1.6 × 1.3 cm and 1.1 × 0.8 cm.

Subsequently, the patient experienced progressive disease (PD), with an increase in the size of the LUL lesion (**Figure 1C**) and emergence of cervical lymph node metastasis (**Figure 3A**). In June 2023, a second LUL biopsy was performed. Unexpectedly, hematoxylin and eosin (HE) staining showed mixed histology of adenocarcinoma and SCLC. Immunohistochemical (IHC) staining confirmed the presence of *thyroid transcription factor-1 (TTF-1)* (weakly +), *napsin A* (+), *synaptophysin* (+), *CD56* (+), and *CgA* (+) (**Figure 2B**). In addition to EGFR 19del, 1012-gene panel testing further demonstrated a TP53 missense mutation, RB1 truncating mutation, EGFR amplification, KIT amplification, and tumor mutational burden (TMB) of 11 mutations per megabase (mt/Mb) (**Table 2**).

### Subsequent treatment regimen and treatment response

**Figure 4:**
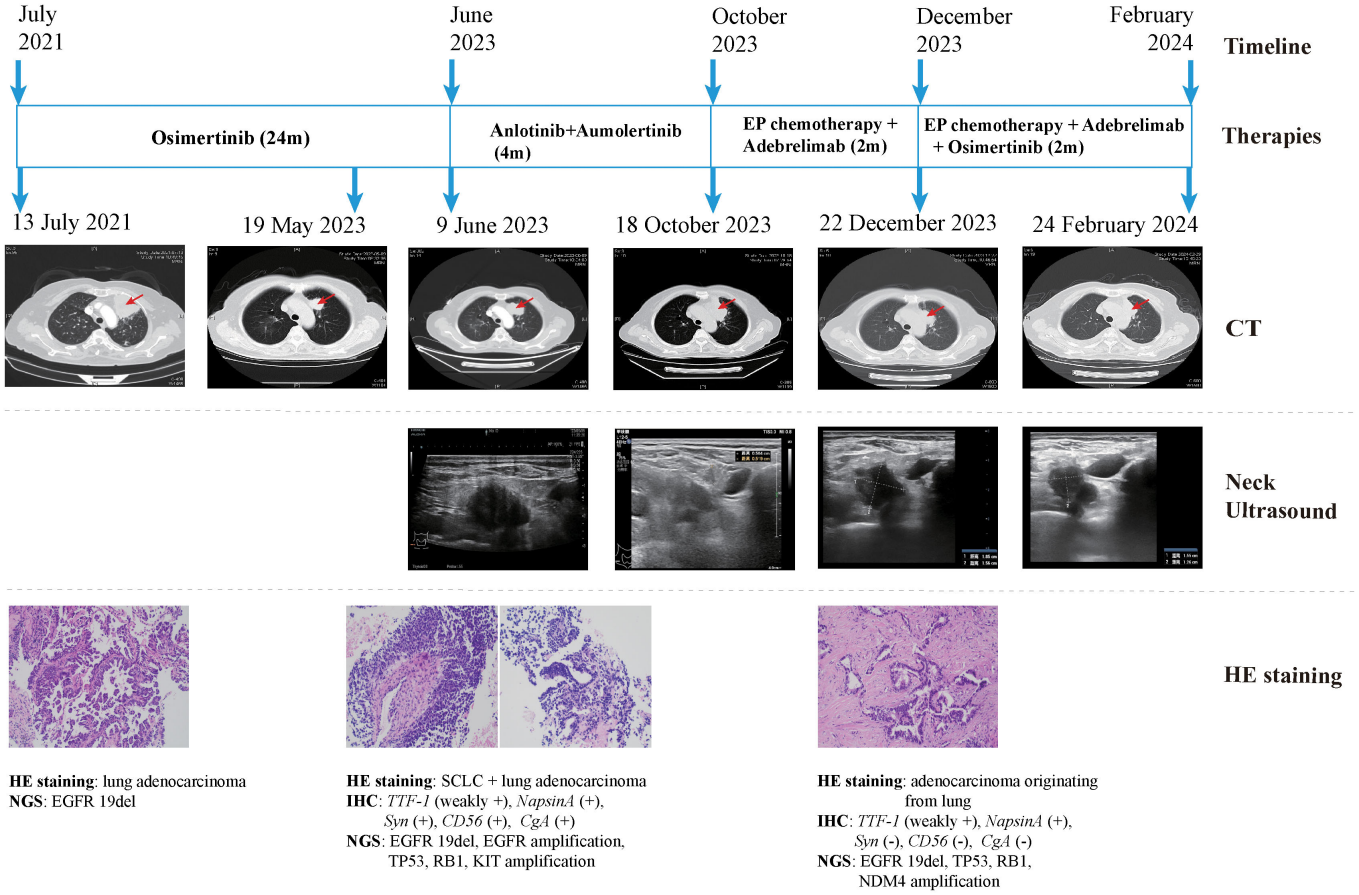
Case presentation of the 68-year-old female patient of SCLC transformation, including timeline, treatment details, chest CT images, neck ultrasound images, HE staining images and NGS findings. CT, computed tomography; HE, hematoxylin and eosin; SCLC, small cell lung cancer; NGS, next-generation sequencing; 19del, exon19 deletion; IHC, immunohistochemistry; *TTF-1*, thyroid transcription factor-1; *Syn*, synaptophysin.

The patient declined the therapeutic option of chemotherapy and instead opted for second-line treatment with a combination of anlotinib and aumolertinib. However, 4 months later, follow-up enhanced CT and neck ultrasonography revealed PD of the LUL lesion (**Figure 1D**) and shrinkage of the cervical lymph nodes (**Figure 3B**). Therefore, the regimen was changed to etoposide plus cisplatin (EP) chemotherapy plus adebrelimab. Following two cycles of EP chemotherapy combined with immunotherapy, the primary lesion located in the LUL exhibited a significant reduction in size (**Figure 1E**), while enlargement of the right cervical lymph node was observed (**Figures 3C, D**). Fine-needle aspiration biopsy of the right cervical lymph node was performed to determine the underlying reasons for the inconsistent response in distinct lesions. Pathological examination revealed poorly differentiated adenocarcinoma originating in the lung (**Figure 2C**). IHC staining demonstrated *TTF-1* (+), *napsin A* (+), *CK7* (+), *synaptophysin* (-), *CD56* (-), and *CgA* (-). 1012-gene panel testing revealed multiple gene mutations, including EGFR 19del, TP53 missense mutation, RB1 truncating mutation, NDM4 amplification, and a TMB of 11 mt/Mb (**Table 2**). Considering the heterogeneity of lung cancer, we introduced osimertinib in addition to the existing chemotherapy and immunotherapy regimens from the third cycle onward. After two cycles of combined treatment, both the primary LUL lesion and metastatic lesion in the cervical lymph nodes showed a notable decrease (**Figures 1F**, **3E, F**). Until the last follow-up in February 2024, no deaths occurred and the follow-up time was 32 months. The flowchart of the treatment process is shown in **Figure 4**.

All procedures performed in this study were in accordance with the ethical standards of the institutional and/or national research committee(s) and the Declaration of Helsinki (as revised in 2013). Written informed consent was obtained from the patient for publication of the case report and accompanying images. A copy of the written consent form is available for review by the journal’s editorial office.

## Discussion

For advanced NSCLC patients with EGFR mutation, the first-line treatment option is EGFR-TKIs, including gefitinib, erlotinib, afatinib, osimertinib, anlotinib, and aumolertinib (34). However, single-agent targeted therapies for NSCLC frequently fail because of the development of acquired drug resistance. Transformation into SCLC represents a rare mechanism of resistance to EGFR-TKIs in advanced lung adenocarcinoma harboring EGFR mutations, accounting for approximately 5-15% of resistance etiologies (35, 36). However, the precise mechanisms underlying this transformation remain unknown. The potential mechanisms of SCLC transformation include epithelial-to-mesenchymal transition (EMT); mutations that affect TP53, RB1, and PIK3CA; and acquired EGFR mutations (35, 37, 38). Patients with a triple-positive mutation profile of EGFR, TP53, and RB1 exhibited a 6-fold augmented susceptibility to SCLC conversion compared with patients without mutations in TP53 and RB1 (39, 40). Few cases of SCLC transformation have been reported in patients receiving immunotherapy, such as programmed death-1 inhibitors (41).

Patients with EGFR-mutated NSCLC who underwent transformation to SCLC exhibited a significantly unfavorable prognosis in terms of survival. A study involving 39 patients reported an average survival duration of merely 6 months after SCLC conversion (42). An analysis of 67 patients revealed a median OS of 10.9 months after SCLC transformation (43). These data imply that timely recognition and efficient intervention play crucial roles in the management of patients undergoing SCLC transformation.

Due to the lack of established treatment guidelines for patients undergoing SCLC transformation, current therapeutic approaches are based on retrospective studies and case reports (6). Platinum and etoposide-based chemotherapy remains the standard treatment for patients with SCLC transformation, with the median disease control time of approximately 3 months. A real-world study included 29 patients who developed SCLC transformation following EGFR-targeted therapy. The analysis indicated that compared to chemotherapy alone, the combination of chemotherapy and targeted therapy improved objective response rates and PFS, although it did not significantly extend OS. Anti-angiogenic therapy and local radiotherapy can prolong OS after transformation (44). A multicenter study involving 32 patients with EGFR-mutant NSCLC who experienced SCLC transformation after targeted therapy revealed that the most commonly used chemotherapy regimen post-transformation was etoposide combined with platinum (n=27), with a median PFS of 3.5 months. Additionally, 3 patients received irinotecan combined with platinum, achieving a median PFS of 7.6 months. Five patients were treated with anlotinib, and the anlotinib group showed a median PFS of 6.2 months (45). Although data suggest that irinotecan combined with platinum and anlotinib may yield better survival outcomes, the limited sample size makes this conclusion less convincing. Furthermore, a case report compared the outcomes of two patients with EGFR-mutant NSCLC who underwent SCLC transformation and received different treatment regimens. One patient received the EP regimen alone post-transformation, achieving a PFS of only 3 months. The other patient received erlotinib combined with the EP regimen, followed by long-term maintenance therapy with erlotinib and oral etoposide, ultimately achieving a PFS of 8 months (46). However, to date, there have been no reports on combined use of chemotherapy, targeted therapy, and immunotherapy for patients with SCLC transformation. In this case, the patient developed PD that transformed into SCLC after 24 months of osimertinib treatment. Further PD occurred following the dual-targeted therapy. Subsequent EP chemotherapy and immunotherapy led to a reduction in the size of the primary lesion and enlargement of cervical lymph nodes. The addition of osimertinib for two cycles resulted in a reduction in both the LUL and cervical lymph node lesions. This finding suggests that EGFR-TKIs only inhibit the EGFR-mutant NSCLC component, allowing the SCLC component to rapidly proliferate and reach PD. EP chemotherapy combined with adebrelimab is the standard treatment for SCLC; thus, simple inhibition of SCLC may lead to rapid regrowth of the NSCLC component. The combination of targeted therapy, chemotherapy, and immunotherapy resulted in a reduction in both primary and metastatic lesions, indicating that mixed histological components of SCLC and NSCLC should be considered. This suggests that for patients experiencing SCLC transformation who still harbor EGFR mutations, a combination of chemotherapy, immunotherapy, and targeted therapy may be an effective treatment approach. However, additional randomized controlled trials are required for further validation. Moreover, recognizing tumor heterogeneity and performing timely biopsies and genetic testing during changes in a patient’s condition are pivotal for facilitating the rapid detection of pathological transformations, tailoring individualized treatment strategies, and enhancing the prognoses of patients.

EGFR-mutated lung adenocarcinoma accompanied by RB1 and TP53 mutations represents the highest-risk group for SCLC transformation during targeted therapy, with a transformation probability of up to 18%. Patients harboring EGFR, RB1, and TP53 mutations exhibit the poorest treatment outcomes, with median time to treatment discontinuation and OS of 9.5 months and 29.1 months, respectively (40). In our case, re-biopsy following disease progression on EGFR-TKIs revealed concurrent EGFR, RB1, and TP53 mutations. Unfortunately, due to the lack of comprehensive genetic analysis at the initial NSCLC diagnosis, only a 14-gene panel was performed, missing critical baseline information on TP53 and RB1 gene status. This underscores the importance of re-biopsy in EGFR/RB1/TP53-mutant lung adenocarcinoma, particularly in patients with poor response to EGFR-TKIs.

In a comprehensive systematic review by Roca et al., 39 patients who underwent SCLC transformation between 2006 and 2016 were systematically evaluated (42). To delve deep into the demographic characteristics, therapeutic interventions, and prognoses of patients experiencing SCLC transformation, we reviewed 33 cases of SCLC transformation from 2017 to 2023 and summarized their genetic mutations, treatment modalities, and patient outcomes in **Table 1**. Among the 33 reported cases, the majority were of Asian ethnicity and demonstrated a pronounced association with poor prognoses, frequently accompanied by central nervous system metastases. Notably, 13 out of 33 patients (39%) presented with central nervous system metastasis. Observational data suggest that male patients (66%) may be more likely to undergo SCLC transformation. What’s more, among the 33 cases, the majority of patients had either an unmentioned family history or no family history, and the patient presented in this case had no history of cancer. It was worth noting that 63% were smokers and 18% were non-smokers, suggesting that smoking may have a potential impact on transformation to SCLC. Disparities in the implementation of personalized medicine across different countries and regions underscore variations in treatment standards and medication accessibility, potentially impacting treatment efficacy and patient survival rates. For instance, Asian populations may prioritize the utilization of the EGFR-TKIs, while Western countries may prioritize the utilization of immunotherapy. EGFR, ALK, and TP53 mutations are commonly observed in patients undergoing SCLC transformation. Among them, EGFR mutations were reported in 13 cases (39%), including 8 cases with EGFR 19 del (62%) and 3 case with EGFR exon 21 L858R (23%). Therefore, we speculate that SCLC transformation is more likely to occur in patients with EGFR mutation and subsequent resistance to targeted therapy.

Surgical specimens were unattainable in patients with unresectable NSCLC at the initial diagnosis. The presence of two histological components could not be definitively excluded because of the inherent limitations of the existing examination methods and techniques. This highlights the importance of obtaining an ample number of tissue specimens from patients with advanced lung cancer to mitigate misdiagnoses resulting from limited sampling.

Despite multiple reported cases of SCLC transformation, treatment strategies remain inadequately explored. In our case report, we document the successful use of EP chemotherapy in combination with adebrelimab and osimertinib for the first time in the management of advanced SCLC transformation. Encouragingly, imaging results indicate a favorable therapeutic response. Nevertheless, the precise molecular mechanism underlying this transformation remains elusive, and consensus treatment guidelines are lacking. Future work should focus on unraveling the molecular mechanisms of this transformation and conducting prospective studies to establish evidence-based treatment protocols.

## Conclusions

SCLC transformation is a rare but crucial cause of acquired EGFR-TKI resistance. It is essential to conduct repeated biopsies and employ NGS and IHC tests to identify alterations in histological types. We found that the combination of EP chemotherapy plus adebrelimab and osimertinib had a significant therapeutic effect in patients with NSCLC pathological transformed to SCLC. The multimodal treatment approach involving chemotherapy, targeted therapy and immunotherapy may be a promising strategy for this distinct patient cohort.

## Data Availability

The original contributions presented in the study are included in the article/supplementary material. Further inquiries can be directed to the corresponding authors.

## Glossary

amp: amplification
AMR: Ceritinib, alectinib, amrubicin
CNS: central nervous system
CT: computed tomography
EMT: epithelial-to-mesenchymal transition
EC: etoposide plus carboplatin
EP: etoposide plus cisplatin
GP: gemcitabine plus cisplatin
HE: hematoxylin and eosin
IC: irinotecan plus carboplatin
IHC: immunohistochemistry
IP: irinotecan plus cisplatin
LUL: left upper lobe
MSS: microsatellite stability
mt/Mb: mutations per megabase
NA: not applicable
NGS: next-generation sequencing
NM: Not mentioned
NSCLC: non-small cell lung cancer
OS: overall survival
PC: pemetrexed plus carboplatin
PP: pemetrexed plus cisplatin
PD: progressive disease
PFS: progression-free survival
PR: partial response
SCLC: small cell lung cancer
Syn: synaptophysin
TC: paclitaxel plus carboplatin
TKI: tyrosine kinase inhibitors
TMB: tumor mutational burden
TTF-1: thyroid transcription factor-1
19del: exon 19 deletion.

## References

[1] Siegel RL Giaquinto AN Jemal A. Cancer statistics, 2024. CA: Cancer J Clin. (2024) 74:12–49. doi: 10.3322/caac.21820 38230766

[2] Lingling X Maoxi C Wei Y Jieting Z Yuanyuan Y Ning X. Transformation of NSCLC to SCLC harboring EML4-ALK fusion with V1180L mutation after alectinib resistance and response to lorlatinib: A case report and literature review. Lung Cancer. (2023) 186:107415. doi: 10.1016/j.lungcan.2023.107415 37907052

[3] Qiao M Li D He Y Zhang C Chi H Li X. Detection and significance of cell-free DNA mutation in pleural effusion in patients with advanced NSCLC. Emergency Med Int. (2022) 2022:3112281. doi: 10.1155/2022/3112281 PMC9205733 35721255

[4] Ohmori T Yamaoka T Ando K Kusumoto S Kishino Y Manabe R. Molecular and clinical features of EGFR-TKI-associated lung injury. Int J Mol Sci. (2021) 22. doi: 10.3390/ijms22020792 PMC7829873 33466795

[5] Herzog BH Devarakonda S Govindan R. Overcoming chemotherapy resistance in SCLC. J Thorac oncology: Off Publ Int Assoc Study Lung Cancer. (2021) 16:2002–15. doi: 10.1016/j.jtho.2021.07.018 34358725

[6] Yin X Li Y Wang H Jia T Wang E Luo Y. Small cell lung cancer transformation: From pathogenesis to treatment. Semin Cancer Biol. (2022) 86:595–606. doi: 10.1016/j.semcancer.2022.03.006 35276343

[7] Imakita T Fujita K Kanai O Terashima T Mio T. Small cell lung cancer transformation during immunotherapy with nivolumab: A case report. Respir Med Case Rep. (2017) 21:52–5. doi: 10.1016/j.rmcr.2017.03.019 PMC5376266 28393006

[8] Oya Y Yoshida T Uemura T Murakami Y Inaba Y Hida T. Serum ProGRP and NSE levels predicting small cell lung cancer transformation in a patient with ALK rearrangement-positive non-small cell lung cancer: A case report. Oncol Lett. (2018) 16:4219–22. doi: 10.3892/ol PMC6126188 30214557

[9] Abdallah N Nagasaka M Abdulfatah E Shi D Wozniak AJ Sukari A. Non-small cell to small cell lung cancer on PD-1 inhibitors: two cases on potential histologic transformation. Lung Cancer (Auckland NZ). (2018) 9:85–90. doi: 10.2147/LCTT PMC6207227 30498383

[10] Liu Y. Small cell lung cancer transformation from EGFR-mutated lung adenocarcinoma: A case report and literatures review. Cancer Biol Ther. (2018) 19:445–9. doi: 10.1080/15384047.2018.1435222 PMC5927699 29461911

[11] Nishioka N Yamada T Harita S Hirai S Katayama Y Nakano T. Successful sequential treatment of refractory tumors caused by small cell carcinoma transformation and EGFR-T790M mutation diagnosed by repeated genetic testing in a patient with lung adenocarcinoma harboring epidermal growth factor receptor mutations: A case report. Respir Med Case Rep. (2018) 25:261–3. doi: 10.1016/j.rmcr.2018.10.004 PMC6176787 30310765

[12] Iams WT Beckermann KE Almodovar K Hernandez J Vnencak-Jones C Lim LP. Small cell lung cancer transformation as a mechanism of resistance to PD-1 therapy in KRAS-mutant lung adenocarcinoma: A report of two cases. J Thorac oncology: Off Publ Int Assoc Study Lung Cancer. (2019) 14:e45–e8. doi: 10.1016/j.jtho.2018.11.031 PMC6382512 30543839

[13] Okeya K Kawagishi Y Muranaka E Izumida T Tsuji H Takeda S. Hyperprogressive disease in lung cancer with transformation of adenocarcinoma to small-cell carcinoma during pembrolizumab therapy. Internal Med. (2019) 58:3295–8. doi: 10.2169/internalmedicine.2892-19 PMC6911749 31327828

[14] Bar J Ofek E Barshack I Gottfried T Zadok O Kamer I. Transformation to small cell lung cancer as a mechanism of resistance to immunotherapy in non-small cell lung cancer. Lung Cancer. (2019) 138:109–15. doi: 10.1016/j.lungcan.2019.09.025 31683093

[15] Miura N Matsubara T Takamori S Haratake N Toyozawa R Yamaguchi M. Histological conversion from adenocarcinoma to small cell carcinoma of the lung after treatment with an immune checkpoint inhibitor: a case report. Oxford Med Case Rep. (2020) 2020:omaa026. doi: 10.1093/omcr/omaa026 PMC7243716 32477576

[16] Si X You Y Zhang X Wang H Wang M Zhang L. Histologic transformation of lung cancer during pembrolizumab therapy: A case report. Thorac Cancer. (2020) 11:793–6. doi: 10.1111/1759-7714.13312 PMC7049493 31944570

[17] Sehgal K Varkaris A Viray H VanderLaan PA Rangachari D Costa DB. Small cell transformation of non-small cell lung cancer on immune checkpoint inhibitors: uncommon or under-recognized? J immunotherapy Cancer. (2020) 8. doi: 10.1136/jitc-2020-000697 PMC7312456 32581048

[18] Arakawa S Yoshida T Shirasawa M Takayanagi D Yagishita S Motoi N. RB1 loss induced small cell lung cancer transformation as acquired resistance to pembrolizumab in an advanced NSCLC patient. Lung Cancer. (2021) 151:101–3. doi: 10.1016/j.lungcan.2020.11.016 33279272

[19] Zhang C Lin L Guo X Chen P. Significance of genetic sequencing in patients with lung adenocarcinoma with transformation to small cell lung cancer: a case report and systematic review. Trans Cancer Res. (2020) 9:3725–33. doi: 10.21037/tcr PMC8798927 35117735

[20] Otoshi R Sekine A Okudela K Asaoka M Sato Y Ikeda S. Small-cell lung carcinoma transformation of lung adenocarcinoma diagnosed by pericardial effusion: A case report. Mol Clin Oncol. (2020) 13:129–32. doi: 10.3892/mco PMC7366234 32714535

[21] Leonetti A Minari R Mazzaschi G Gnetti L La Monica S Alfieri R. Small cell lung cancer transformation as a resistance mechanism to osimertinib in epidermal growth factor receptor-mutated lung adenocarcinoma: case report and literature review. Front Oncol. (2021) 11:642190. doi: 10.3389/fonc.2021.642190 33981604 PMC8107466

[22] Imakita T Fujita K Kanai O Okamura M Hashimoto M Nakatani K. Small cell transformation of non-small cell lung cancer under immunotherapy: Case series and literature review. Thorac Cancer. (2021) 12:3062–7. doi: 10.1111/1759-7714.14180 PMC8590890 34622569

[23] Zhai X Liu J Liang Z Li Z Liu Y Huang L. Case report: re-sensitization to gefitinib in lung adenocarcinoma harboring EGFR mutation and high PD-L1 expression after immunotherapy resistance, which finally transform into small cell carcinoma. Front Oncol. (2021) 11:661034. doi: 10.3389/fonc.2021.661034 34249697 PMC8264361

[24] Yang Z Lin Y Wang H. Transformation of non-small cell lung cancer into small cell lung cancer in a patient with advanced lung cancer: a case report. J Int Med Res. (2021) 49:3000605211035005. doi: 10.1177/03000605211035005 34396834 PMC8371742

[25] Li YC. Durable response to durvalumab-based immunochemotherapy in small-cell lung carcinoma transformation from EGFR-mutant non-small cell lung cancer: A case report. Thorac Cancer. (2022) 13:775–9. doi: 10.1111/1759-7714.14325 PMC8888151 35088537

[26] Liu H Chen LH Zhang ZH Wang N Zhuang SH Chen H. Histomorphological transformation from non-small cell lung carcinoma to small cell lung carcinoma after targeted therapy or immunotherapy: A report of two cases. Front Oncol. (2022) 12:1022705. doi: 10.3389/fonc.2022.1022705 36439460 PMC9683475

[27] Yang MH Yu J Cai CL Li W. Small cell lung cancer transformation and tumor heterogeneity after sequential targeted therapy and immunotherapy in EGFR-mutant non-small cell lung cancer: A case report. Front Oncol. (2022) 12:1029282. doi: 10.3389/fonc.2022.1029282 36568150 PMC9768476

[28] Fang G Liu W Shang Y Huo R Shi X Wang Y. Characterization of non-small cell lung cancer transforming to small cell lung cancer and its response to EGFR-TKI: a case report. Ann Trans Med. (2022) 10:115. doi: 10.21037/atm PMC8848416 35282065

[29] Wang D Ye W Chen D Shi Q Ma D. Transformation of lung squamous cell carcinoma to small cell lung cancer after immunotherapy resistance: A case report. Cancer Manage Res. (2023) 15:803–8. doi: 10.2147/CMAR.S420485 PMC10424693 37583652

[30] Wang X Liang J Li L Pan Z Wang L. Reuse of osimertinib after small cell lung cancer transformation in lung adenocarcinoma with *de-novo* epidermal growth factor receptor T790M mutation: case report. Anti-cancer Drugs. (2023) 34:306–10. doi: 10.1097/CAD.0000000000001403 36206142

[31] Gazeu A Aubert M Pissaloux D Lantuejoul S Pérol M Ikhlef N. Small-cell lung cancer transformation as a mechanism of resistance to pralsetinib in RET-rearranged lung adenocarcinoma: A case report. Clin Lung Cancer. (2023) 24:72–5. doi: 10.1016/j.cllc.2022.10.005 36437214

[32] Peng Y Zheng Z Zewen W Yanan L Mingyan Z Meili S. Whole-exome sequencing explored mechanism of selpercatinib resistance in RET-rearranged lung adenocarcinoma transformation into small-cell lung cancer: a case report. BMC pulmonary Med. (2023) 23:492. doi: 10.1186/s12890-023-02799-5 38057798 PMC10698965

[33] Xia G Huang J Ni J Song M Zhang J Hofman P. Transformation of ALK-positive NSCLC to SCLC after alectinib resistance and response to combined atezolizumab: a case report. Trans Lung Cancer Res. (2023) 12:637–46. doi: 10.21037/tlcr PMC10088007 37057117

[34] Oshima Y Tanimoto T Yuji K Tojo A. EGFR-TKI-associated interstitial pneumonitis in nivolumab-treated patients with non-small cell lung cancer. JAMA Oncol. (2018) 4:1112–5. doi: 10.1001/jamaoncol.2017.4526 PMC5885195 29327061

[35] Lee JK Lee J Kim S Kim S Youk J Park S. Clonal history and genetic predictors of transformation into small-cell carcinomas from lung adenocarcinomas. J Clin oncology: Off J Am Soc Clin Oncol. (2017) 35:3065–74. doi: 10.1200/JCO.2016.71.9096 28498782

[36] Oser MG Niederst MJ Sequist LV Engelman JA. Transformation from non-small-cell lung cancer to small-cell lung cancer: molecular drivers and cells of origin. Lancet Oncol. (2015) 16:e165–72. doi: 10.1016/S1470-2045(14)71180-5 PMC4470698 25846096

[37] Sequist LV Waltman BA Dias-Santagata D Digumarthy S Turke AB Fidias P. Genotypic and histological evolution of lung cancers acquiring resistance to EGFR inhibitors. Sci Trans Med. (2011) 3:75ra26. doi: 10.1126/scitranslmed.3002003 PMC3132801 21430269

[38] Yu HA Arcila ME Rekhtman N Sima CS Zakowski MF Pao W. Analysis of tumor specimens at the time of acquired resistance to EGFR-TKI therapy in 155 patients with EGFR-mutant lung cancers. Clin Cancer research: an Off J Am Assoc Cancer Res. (2013) 19:2240–7. doi: 10.1158/1078-0432.CCR-12-2246 PMC3630270 23470965

[39] Shaurova T Zhang L Goodrich DW Hershberger PA. Understanding lineage plasticity as a path to targeted therapy failure in EGFR-mutant non-small cell lung cancer. Front Genet. (2020) 11:281. doi: 10.3389/fgene.2020.00281 32292420 PMC7121227

[40] Offin M Chan JM Tenet M Rizvi HA Shen R Riely GJ. Concurrent RB1 and TP53 Alterations Define a Subset of EGFR-Mutant Lung Cancers at risk for Histologic Transformation and Inferior Clinical Outcomes. J Thorac oncology: Off Publ Int Assoc Study Lung Cancer. (2019) 14:1784–93. doi: 10.1016/j.jtho.2019.06.002 PMC6764905 31228622

[41] Hsu PC Jablons DM Yang CT You L. Epidermal growth factor receptor (EGFR) pathway, yes-associated protein (YAP) and the regulation of programmed death-ligand 1 (PD-L1) in non-small cell lung cancer (NSCLC). Int J Mol Sci. (2019) 20. doi: 10.3390/ijms20153821 PMC6695603 31387256

[42] Roca E Gurizzan C Amoroso V Vermi W Ferrari V Berruti A. Outcome of patients with lung adenocarcinoma with transformation to small-cell lung cancer following tyrosine kinase inhibitors treatment: A systematic review and pooled analysis. Cancer Treat Rev. (2017) 59:117–22. doi: 10.1016/j.ctrv.2017.07.007 28806542

[43] Marcoux N Gettinger SN O’Kane G Arbour KC Neal JW Husain H. EGFR-mutant adenocarcinomas that transform to small-cell lung cancer and other neuroendocrine carcinomas: clinical outcomes. J Clin oncology: Off J Am Soc Clin Oncol. (2019) 37:278–85. doi: 10.1200/JCO.18.01585 PMC7001776 30550363

[44] Wang S Xie T Hao X Wang Y Hu X Wang L. Comprehensive analysis of treatment modes and clinical outcomes of small cell lung cancer transformed from epidermal growth factor receptor mutant lung adenocarcinoma. Thorac Cancer. (2021) 12:2585–93. doi: 10.1111/1759-7714.14144 PMC8487822 34490724

[45] Wang W Xu C Chen H Jia J Wang L Feng H. Genomic alterations and clinical outcomes in patients with lung adenocarcinoma with transformation to small cell lung cancer after treatment with EGFR tyrosine kinase inhibitors: A multicenter retrospective study. Lung Cancer. (2021) 155:20–7. doi: 10.1016/j.lungcan.2021.03.006 33714778

[46] Lai L Meng W Wei J Zhang X Tan Z Lu Y. Transformation of NSCLC to SCLC after 1st- and 3rd-generation EGFR-TKI resistance and response to EP regimen and erlotinib: 2 CARE-compliant case reports. Med (Baltimore). (2021) 100:e25046. doi: 10.1097/MD.0000000000025046 PMC7969239 33725888

